# Stigmatizing attitude of psychiatrists in the Netherlands

**DOI:** 10.1192/j.eurpsy.2024.353

**Published:** 2024-08-27

**Authors:** S. Kakar, T. Birkenhäger, L. Baars, D. Őri

**Affiliations:** ^1^Department of Psychiatry, Erasmus University Medical Center, Rotterdam; ^2^Department of Mentalization Based Treatment in Adults, Viersprong Institute for Studies on Personality Disorder, Bergen op Zoom, Netherlands; ^3^Institute of Behavioural Sciences, Semmelweis University; ^4^Department of Mental Health, Heim Pal National Pediatric Institute, Budapest, Hungary

## Abstract

**Introduction:**

Even in the current times people with mental health disorders face negative treatment due to negative stereotyping. This occurs not only within their private environment and in the public community, but also by healthcare professionals. Mental health related stigma results in various disadvantages, such as: worse treatment in healthcare and discrimination in job interviews, in work environment, in education and in housing.

**Objectives:**

Our aim with this cross-sectional study, was to investigate the attitudes of adult and child psychiatrists in the Netherlands towards people with mental health problems.

**Methods:**

We used the Opening Minds Stigma Scale for Health Care Providers (OMS-HC) to measure the stigmatizing attitudes. Participants filled in this internet-based survey anonymously. The OMS-HC total scores as well as the subscales were used to determine the stigma.

**Results:**

Altogether, N=170 practitioners (n=45 males, n=124 females) completed the survey. The bifactor ESEM model showed the best model fit (RMSEA=0.057, CFI=0.968, TLI=0.935); however, exploratory factor analysis results indicated the weakness of items 13 and 15. Participants who provide psychotherapy to their patients prefer less social distance towards them (9(7-10) vs 10(7.5-11), p=0.051)). Also those who have ever been treated medically for their own mental health problems, prefer less social distance (7,5(6-10) vs 9(8-11), p=0.009). Rural working psychiatrists are more willing to disclose and seek help for their mental health problems than those working in urban areas (9 (8-10) vs 8 (6.5-9.5), p = 0.024). Those who are open to (29(26-32.5) vs 32.5(31.25-35), p=0.009) or having an opportunity to regularly participate in case discussion groups (29(25.25-32) vs 32(28-35.25), p=0.012) have an overall favourable attitude towards people with mental health problems.

**Conclusions:**

This is the first study on the stigmatizing attitude of practicing psychiatrists in the Netherlands from their own perspectives. It will contribute to the gaps of knowledge of the stigmatizing attitude of psychiatrists towards people with mental health problems. Moreover this study will provide new interventions towards less stigmatizing attitude of psychiatrists.

**Disclosure of Interest:**

None Declared

